# Erratum for Mahazu et al., “Klebsiella Species and Enterobacter cloacae Isolates Harboring *bla*_OXA-181_ and *bla*_OXA-48_: Resistome, Fitness Cost, and Plasmid Stability”

**DOI:** 10.1128/spectrum.00521-23

**Published:** 2023-02-21

**Authors:** Samiratu Mahazu, Isaac Prah, Yusuke Ota, Takaya Hayashi, Yoko Nukui, Masato Suzuki, Yoshihiko Hoshino, Yukihiro Akeda, Toshihiko Suzuki, Tomoko Ishino, Anthony Ablordey, Ryoichi Saito

## ERRATUM

Volume 10, no. 6, e03320-22, 2022, https://doi.org/10.1128/Spectrum.03320-22. Page 4, line 2: “in the chromosome” should read “on the chromosome”. Page 10, “Plasmid transfer experiments” paragraph line 1: “IncL-*bla*_OXA-181_” should read “IncL-*bla*_OXA-48_”. Pages 12 and 13: Reference entries 15 through 22 should be numbered 39 through 46, respectively, and reference entries 23 through 46 should be numbered 15 through 38, respectively.

